# Knowledge, Attitudes, and Practices of the Saudi Arabian Population Regarding Contaminated Banknotes: Implications for Infectious Disease Transmission and Analyzing the Biofilm in Wallet as a Reservoir

**DOI:** 10.1155/cjid/4611971

**Published:** 2025-05-22

**Authors:** Mashael Almogbel, Mohsina Huq, Meshal Almogbel, Ahmad Almatroudi, Khaled S. Allemailem

**Affiliations:** ^1^Department of Medical Laboratories, College of Applied Medical Sciences, Qassim University, Buraydah 51452, Saudi Arabia; ^2^College of Medicine, Qassim University, Buraydah 51452, Saudi Arabia

**Keywords:** attitude, biofilm, contaminated currency notes, knowledge, MDR, practice, Saudi Arabia

## Abstract

**Introduction:** Contaminated paper currency may serve as a potential source for multidrug-resistant pathogens, posing risks not only to individuals who handle cash but also to public health. This study aimed to evaluate the knowledge, attitudes, and practices (KAP), and microbial contamination of paper currency and biofilm formation in the wallet as a reservoir of contamination in Saudi Arabia (KSA).

**Methods:** Data were collected through an online survey assessing the KAP of the Saudi population regarding the use of contaminated notes across various provinces from February to April 2018. The analysis was conducted using EPI INFO V7 software. Microorganisms were isolated and identified from paper and plastic currency collected from slaughterhouses, gas stations, and hospital cafeterias. The MicroScan WalkAway system was utilized for confirmation and antimicrobial resistance (AMR) testing, while scanning electron microscopy (SEM) was employed to visualize biofilms present in wallets.

**Results:** Among the 1415 adult Saudi citizens surveyed, 75% lacked awareness about contaminated currency. Over 50% reported not washing their hands after handling contaminated notes, with 78% of those being male. Fifteen different microbial species were isolated from contaminated notes, including *Staphylococcus* and fecal coliforms. Multidrug-resistant *Staphylococcus* and *Enterobacter* were detected in nearly all paper notes, while extended-spectrum beta-lactamase (ESBL) *E*. *coli* was found only in 50-riyal notes. Plastic notes showed no bacterial contamination. SEM images of the interior surfaces of wallets revealed the presence of extracellular polymeric substances (EPSs) in biofilms, along with cocci-shaped bacteria.

**Conclusion:** To mitigate health risks, it is recommended that paper notes be replaced with plastic currency, and efforts should be made to raise awareness among the Saudi population regarding the dangers posed by contaminated notes.

## 1. Introduction

Paper currency is repeatedly exchanged for goods and services [1]. As a result, its circulation from person to person presents a significant risk for spreading microorganisms, making it a potential vector for disease transmission [2]. Paper currency contaminated with pathogenic bacteria may contribute to increased infection and mortality rates [3–5]. Communicable diseases can spread via fomites, and the transfer of pathogens through paper currency is a plausible route, especially since these currencies, typically made from cotton and linen, can harbor various harmful microorganisms [6].

Contaminated paper currency can be a significant source of disease transmission, especially if it carries pathogenic microorganisms resistant to common antibiotics, posing a public health risk [4]. In Saudi Arabia, 88% of currency notes were found to be contaminated with various microorganisms [7]. This highlights the likelihood of banknotes acting as reservoirs and vehicles for the spread of multidrug-resistant (MDR) organisms. A study conducted in Riyadh, Saudi Arabia, investigated bacterial and fungal contamination on money and cell phones, identifying fungi such as *Aspergillus niger*, *Aspergillus flavus*, *Candida* spp., *Penicillium* spp., and *Rhizopus* spp., and bacteria including *Klebsiella* spp., *Staphylococcus* spp., *Enterobacter cloacae*, *E*. *coli*, and *Yersinia pseudotuberculosis* [8]. Lower denomination bills harbor the highest concentration of infectious agents because they are exchanged more frequently [9]. In the United States, 94% of $1 bills were found to be contaminated with bacteria [6], while 80 ± 5% of old two-taka notes in Bangladesh were contaminated with coliforms [10]. In Nigeria, 89% of Naira notes were found to be contaminated with bacteria [11]. An Australian study involving currencies from 10 countries found that lower denomination notes tend to have higher bacterial content, which is influenced by the age of the notes and the production material [12]. A European study involving 12 countries showed that while people were aware of dirty cash, they rarely washed their hands after handling contaminated notes, though they recognized the health risks [13].

Currency is handled by individuals with varying levels of hygiene, from different occupations, and stored in different environments. Unhygienic practices such as placing money inside the blouse, in socks, or shoes, or squeezing it in hands introduce microbes to the notes [14]. Wetting fingers with saliva to count money or handling notes with food-contaminated hands further promotes contamination and increases the risk of infection [11]. A lack of awareness and public health policies regarding the handling of currency may contribute to the spread of infectious diseases, which are exacerbated by factors like population growth, urbanization, poverty, and even bioterrorism. People need to understand the risks associated with handling contaminated paper currency and the importance of proper hand hygiene.

However, there are very few studies in Saudi Arabia regarding the KAP and relation between infection transmission from contaminated notes. There are no specific studies on the inside condition of a wallet in use, whether the wallet is serving as a continuous source of microbiota contamination to the notes. Therefore, this study aims to evaluate the knowledge, attitudes, and practices (KAP) of the Saudi population regarding contaminated currency notes and assess the microbial contamination of paper currency circulating in Saudi Arabia. We also investigated whether the wallet acts as a potential reservoir of microorganisms by forming biofilm inside.

## 2. Methodology

We conducted both an observational and experimental study using a cross-sectional study design. The observational data were gathered through an online survey using a structured questionnaire that assessed the KAP of the Saudi population regarding currency notes. The study was conducted across various provinces and regions of Saudi Arabia from February 2018 to April 2018. For microbial isolation, currency notes were collected from all Saudi denominations from different locations in Qassim. A scanning electron microscope (SEM) was used to observe biofilm development on samples, such as those stored in wallets.

### 2.1. Study Participants

The observational study was conducted across several provinces and regions of Saudi Arabia. A sample size of 1415 respondents, all Saudi nationals aged 18 and above, participated in the survey.

### 2.2. Data Collection

Data were collected via an online survey/questionnaire (Supporting Appendix 1). The questionnaire was not pre-tested before conducting the survey. Invitations were disseminated through a URL link published on various websites and media platforms. Traditional recruitment methods, such as sending email invitations and encouraging participants to invite friends and family, were also used. Snowball sampling, starting with a small number of respondents and expanding as participants invited others, increased the sample size. The survey questions were initially developed in English and then translated into Arabic to accommodate different age groups and educational levels within the Saudi population. The questions were simple, direct, and designed to assess the KAP of adult Saudis regarding contaminated currency. The survey took approximately 5–10 min to complete, and it consisted of self-administered, open, and close-ended questions with multiple-choice options, providing both qualitative and quantitative data. Informed consent was obtained from participants, and all data were kept confidential and used solely for research purposes.

### 2.3. Study Sample Collection

Samples were collected between January 16, 2021, and November 2, 2021. Currency notes were gathered from all Saudi denominations (1, 5, 10, 50, 100, and 500 SAR) from seven different locations, including slaughterhouses, gas stations, hospital cafeterias, grocery stores, food refrigerators, restaurants, and vegetable markets in Qassim. These locations were randomly selected, and two samples were taken from each currency category at each site. For the five-riyal note, samples were collected from both plastic-based and cotton-based versions.

In total, 98 notes were collected from the Al Qassim region. All currency samples were obtained randomly and aseptically. Sterile gloves were used to handle the notes, and they were stored in sterile containers. A sterile, cotton-tipped swab moistened with sterile physiological saline was used to swab both sides of the notes.

### 2.4. Sample Processing

Once collected, the swabs were labeled and transported to the lab, where they were incubated with tryptic soy broth (TSB) with glycerol for 1 h to recover any stressed or weak microorganisms before culturing on blood agar, MacConkey agar, and chocolate agar. The cultures were incubated aerobically at 35°C–37°C for 24–48 h. Isolated colonies were identified and characterized using the MicroScan WalkAway 96 Pulse System (Beckman Coulter, Inc., USA) for both identification and antimicrobial susceptibility testing.

The SEM was used to visualize biofilm formation in wallet samples, which were collected from various individuals. Wallet samples (up to 1 cm^2^) were fixed in 3% glutaraldehyde, dried in ethanol, and then immersed in hexamethyldisilazane (HMDS, Polysciences, Inc., Warrington, PA, USA) for 3 minutes. After sputter-coating with a 20-nm gold film, the samples were examined by SEM.

### 2.5. Data Analysis

The data were entered into Microsoft Excel 2007 and analyzed using EPI INFO 7 software. Descriptive statistics, including frequency and percentages for categorical variables and mean/standard deviation for continuous variables, were used to analyze the data. Associations between outcome and predictor variables were tested using Chi-square analysis, with the level of statistical significance set at 5%.

## 3. Results

A total of 1415 Saudi adult citizens participated in this online survey after providing informed consent. The majority of participants were young and educated, with most being between the ages of 18 and 29 (Table 1). Geographically, the highest representation came from Al Qassim (57%), followed by Riyadh (20%) and the Eastern Province (6.75%), with participants from other regions making up less than 5%.

In terms of occupation, most respondents were schoolteachers, followed by university professors, government employees, lab specialists, administrators, and managers. Other professions included financial officers, lawyers, soldiers, dietitians, social specialists, secretaries, salespeople, nurses, programmers, engineers, dentists, mechanics, translators, veterinarians, bio-researchers, businesswomen, and caregivers. From the survey, 59.51% of participants were unaware of the level of contaminants on Saudi currency notes, and 75.90% had not received any information regarding contaminated currency notes or their sources (Table 1).

**Figure 1:**
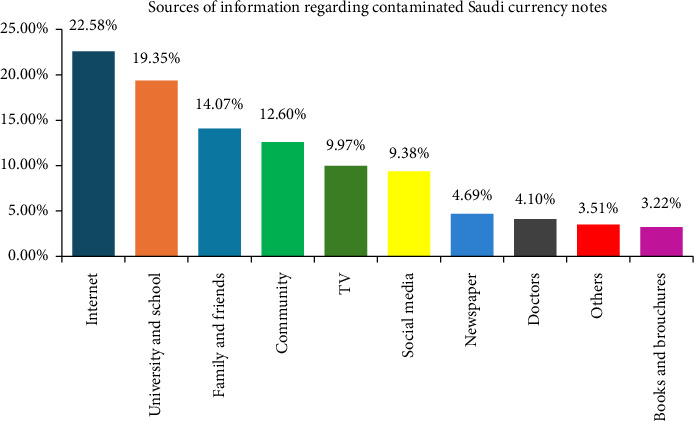
Distribution of sources of information regarding contaminated currency notes among study participants.

The most common sources of information about contaminated currency notes among respondents (*N* = 341) were the Internet, followed by universities and schools, then family, friends, and the broader community (Figure 1). This indicates that the respondents knew because contaminated currency notes are getting an interest from all populations in different age groups. Eighty-seven percent of participants correctly identified contaminated currency notes (Table 1). However, 13.07% selected incorrect options, with some choosing both contaminated and uncontaminated notes, while others failed to recognize the contaminated notes and chose only the clean ones.

**Figure 2:**
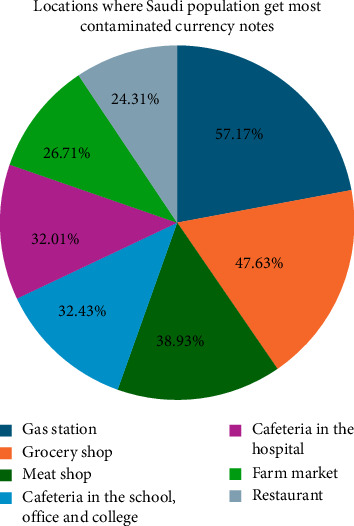
Distribution of various locations from where the Saudi population get contaminated currency notes.

Nearly 87% of the Saudi population was unaware of the appropriate actions to take with contaminated currency notes, while only 13.43% understood how to handle them. Of those informed, 4.78% would exchange contaminated notes at the bank, 48.40% would sterilize both their hands and the currency, 16.48% would wash and press the contaminated notes, and 17.02% would get rid of them by purchasing items or returning them to stores. Additionally, 5.85% would store the contaminated notes in their bags or wallets, 4.25% would continue using them despite the contamination, and 1.59% would either burn or tear up the contaminated notes. Gas stations were the most common source of contaminated currency, followed by grocery stores and meat shops, while other locations had similar contamination levels (Figure 2). Only 7.77% of respondents correctly identified all possible sources of contaminated currency, acknowledging that it can be acquired from any of these places.

**Figure 3:**
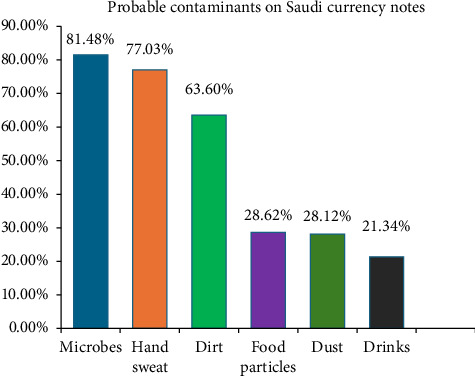
Distribution of the probable contaminants on Saudi currency notes as reported by study participants.

The most perceived contaminants on currency notes among the Saudi population are microbes, followed by hand sweat and dirt. Other contaminants were reported at similar percentages. Interestingly, only 9.25% of respondents selected all the contaminants, which is the correct answer since contaminated currency notes can harbor all the mentioned substances (Figure 3). In an open-ended question, respondents listed additional potential contaminants, including blood, nasal mucus, sneezing, coughing, infectious diseases, urine, feces, makeup, pesticides, saliva, ink, ear secretions, heroin, contaminated water, automobile oil, dyes, and henna.

The data revealed that 57.06% of the Saudi population did not wash their hands after handling contaminated currency notes, while 42.94% did wash their hands and provided details on their handwashing practices (Table 2). Additionally, 65.18% of respondents believed that contaminated currency notes could transmit illnesses such as gastrointestinal issues, sore throats, acne, blisters, diarrhea, and eye allergies. In contrast, 34.82% did not think that contaminated currency notes were likely sources of disease transmission (question no. 16). Moreover, a significant majority (94.53%) of participants agreed that the Saudi community needs greater knowledge and awareness regarding contaminated currency notes, while 5.47% did not share this view. Data on the storing practice of currency in the Saudi population were collected, where it was found that 90% thought that storing money in unusual places like under the carpet or inside clothes, socks, or shoes was not acceptable (Table 3).

Significant differences were observed between male and female participants in their attitudes toward handling contaminated currency (Table 4). Employment status also played a key role in shaping attitudes, particularly regarding the handling, counting, and storage of contaminated currency (Table 4). Employed individuals demonstrated greater caution when dealing with money, such as washing their hands after handling dirty currency and during the counting process (Table 5).

**Figure 4:**
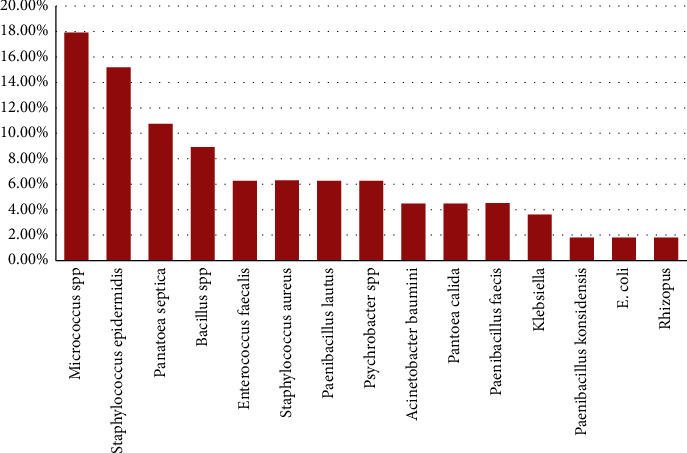
Types of microorganisms isolated from different currency notes.

Both Gram-positive and Gram-negative bacteria were isolated and identified using the MicroScan WalkAway 96 pulse system. The most commonly isolated organisms were Gram-positive, including *E*. *faecalis*, *S*. *aureus*, *Micrococcus* spp., *S*. *epidermidis*, and *Bacillus* spp., *Paenibacillus lautus*. Among Gram-negative bacteria, we identified *E*. *coli*, *Klebsiella*, *Panatoea septica*, *P*. *calida*, and *Acinetobacter baumannii* (Figure 4). Plastic five currency notes showed no bacterial growth.

The antimicrobial susceptibility test revealed that Gram-positive bacteria exhibited higher rates of multidrug resistance compared to Gram-negative bacteria (Tables 6 and 7). Among the Gram-negatives, *E*. *coli* was the most frequently detected, followed by *Pseudomonas* spp., *Enterobacter*, *Acinetobacter* spp., and *Klebsiella* spp., with *E*. *coli* found on all types of currency. Higher-denomination notes harbored more organisms overall. A total of 60 Gram-negative isolates were detected, of which 68% were MDR. Among the Gram-positives, *Enterococcus* and *Staphylococcus* spp. were more frequently isolated, with *Staphylococcus* species being the most common. Of the 24 Gram-positive isolates, 91.6% were MDR.

Currency notes were collected from seven different locations (Table 8) where money is frequently exchanged. Gas stations harbored the highest number of microorganisms, both Gram-negative and Gram-positive, followed by restaurants and hospital cafeterias. Notably, 100% of the Gram-negative organisms from hospital cafeterias and grocery shops were MDR. While fewer Gram-positive organisms were isolated, the majority of them were also found to be MDR.

**Figure 5:**
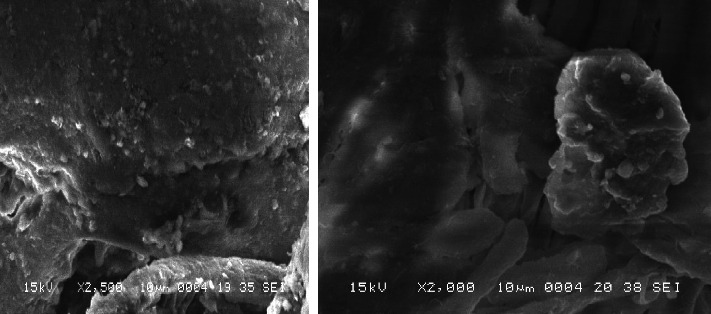
SEM pictures of inside the money wallet surface, showing extracellular polymeric substance of biofilm and cocci bacteria.

SEM revealed notable findings. We examined a section of a leather wallet and discovered a developed biofilm. This investigation was prompted by survey data showing that 76.39% of participants stored their money in wallets. Since direct microscopy of currency notes was not allowed, we instead examined small wallet samples collected from different individuals. Two of these samples tested positive for biofilm, with bacteria observed adhering to surfaces and encased in extracellular polymeric substances (EPSs) (Figure 5).

## 4. Discussion

The data for this study were gathered through an online survey from 1415 Saudi adult citizens across various provinces and regions of Saudi Arabia. The survey focused on the KAP of participants regarding the use of contaminated currency notes. The majority of respondents were aged 18–29, representing a young, educated population that is particularly interested in raising awareness about contaminated currency. This age group also aligned well with the study's measurement needs. Notably, many participants worked as schoolteachers, a group considered highly influential. Teachers in direct contact with young students, from children to adolescents in high school, often serve as role models. If teachers lack sufficient knowledge about contaminated currency and how to handle it properly, it could negatively impact students' health, especially younger children, increasing the risk of spreading infectious diseases within the community.

Contaminated currency notes are a global concern because they pass between individuals of different age groups, hygienic practices, and immune conditions. People living in poor sanitary environments or practicing inadequate hygiene can contaminate currency with microorganisms. This often occurs through improper hand washing after using the toilet, licking fingers to count bills, coughing or sneezing into hands before handling money, and placing or storing currency on dirty surfaces. These contaminated notes then act as vehicles, transferring microorganisms to the hands of the next user. As a result, the circulation of paper currency can facilitate the spread of infectious agents. Currency is an effective vector for disease transmission [15], and in Saudi Arabia, contamination levels of paper notes have been found to reach as high as 88% [6].

A significant portion of the Saudi population is unaware of contaminated currency notes. Among those who have heard about it (*N* = 341), the Internet was cited as the most common source of information. The second most common source was universities and schools, indicating some efforts to raise awareness in the educational sector. However, there remains a need to further increase knowledge and awareness about contaminated currency in educational institutions for betterment of the Saudi population. A large portion of respondents lacked sufficient KAP regarding contaminated currency notes. To assess the relationship between responses and various demographic factors, the data were categorized by gender, age, education level, and employment. The findings revealed that gender was a significant factor influencing responses related to KAP concerning contaminated currency. Females scored higher than males across all measures, possibly due to the higher number of female respondents and personal hygiene.

In terms of knowledge, most respondents were unaware of the level of contaminants on Saudi currency notes, with males being less informed than females. Surprisingly, those with higher education levels were less knowledgeable compared to those with lower education levels, suggesting a lack of sufficient awareness about contaminated currency in educational programs. Additionally, the majority of respondents did not know how to handle contaminated currency notes. However, most participants were able to correctly identify contaminated notes from a set of images, indicating some ability to differentiate between clean and contaminated currency.

A study conducted in Europe found that 27% of Europeans never wash their hands after touching money [13]. In contrast, our study revealed a much higher percentage (57%) of Saudis who reported not washing their hands after handling contaminated currency, with the majority being male (78%). Among those who did wash their hands (43%), 48% were female, explaining that they used water and soap. This indicates a negative practice within the Saudi population regarding hand hygiene after handling currency. In Europe, 64% of people believe cash is dirtier than public transport, door handles, communal food, and vending machines, while 65.18% of the Saudi population in our study recognized that contaminated currency notes could transmit infectious diseases, demonstrating a generally positive attitude toward awareness of the risks. A study conducted in Mokhada, during the 2016–2017 school year, found that 62% of people obtained clean currency notes from ATMs, 36% from banks, 92% wet their hands with saliva while handling money, and 100% never washed their hands after doing so [16].

In our study, only 4.78% of Saudi respondents knew how to properly handle contaminated currency notes, and 13.43% would replace them at banks. Some participants mentioned they would destroy, wash, or return the contaminated notes to a vendor to get rid of them. Notably, 97% did not use saliva when counting notes, but 57% did not wash their hands afterward. While some practices were more positive compared to other study [16], overall, the Saudi population demonstrated negative practices and requires greater awareness about handling contaminated currency.

Most respondents in our study preferred using their fingers rather than saliva when counting bundles of currency notes. Girma [17] noted that many people, especially market women, motorcyclists, bus drivers, conductors, butchers, and restaurant operators, do not carry money in wallets, often squeezing paper currency instead. However, our study found that most respondents stored their currency notes in wallets, followed by bags, inside clothing pockets, treasuries, drawers, and other unconventional places like under couches, inside books, or between makeup products. Although using a wallet is common, it is not ideal, as the close proximity to body heat can promote microbial growth. The safest places to store money would be in banks or credit cards, which should be prioritized, while only some of the money should be kept in wallets.

Our analysis of microbial contamination on paper currency revealed mostly bacterial isolates and one strain of fungus, *Rhizopus* spp. Similarly, a study conducted in Riyadh found that 72.3% of the currency was contaminated with pathogenic bacterial and fungal strains [8]. Both Saudi currency notes and mobile phones are highly contaminated with enteric bacteria and fungi, many of which can cause diseases in humans. To reduce the transmission of infections from contaminated paper currencies and mobile phones, a robust public health campaign should be implemented. This would emphasize the importance of proper hand washing after handling money. Without such interventions, the rate of infection and mortality from these pathogens could continue to increase. This study surveyed bacterial and fungal contamination on paper money and mobile phones, identifying the most common fungal species as *Aspergillus niger*, followed by *A*. *flavus*, *Candida* spp., *Penicillium* spp. and *Rhizopus* spp. [8].

In our study, we examined both paper and plastic currency notes. In Saudi Arabia, paper currency is widely used, from small shops to large malls. Shopping and dining are popular activities, and while most shopping centers provide well-maintained restrooms, not everyone practices hand washing. Additionally, even the use of hand sanitizers does not fully eliminate harmful pathogens [18]. A study in Jeddah investigating bacterial contamination on the one Riyal paper note found that the more frequently a bill was handled, the greater its contamination. Eighty-eight percent of notes were contaminated with mixed bacterial growth (≥ 2 types), with older notes showing Gram-positive bacilli (79%), coagulase-negative staphylococci (75%), *Staphylococcus aureus* (38%), *Klebsiella* spp. (21%), *Pseudomonas* spp. (19%), *Escherichia coli* (9%), viridans group streptococci (VGS) (8%), and non-hemolytic streptococci (4%). Newer notes also showed mixed bacterial contamination but at lower rates [7].

A study conducted in Riyadh in 2017 found a higher occurrence of Gram-positive bacteria compared to that of Gram-negative, where *Bacillus* sp. (56.84%), *Staphylococcus* sp. (25.03%), *Klebsiella* sp. (13.40%), and *E*. *coli* (4.71%) were detected on all currency notes studied [19]. In Nigeria, bacterial contamination, including *E*. *coli*, *Klebsiella* spp., and *Staphylococcus* spp., were found on currency notes collected from food vendors [20]. In our study, however, we found a higher proportion of Gram-negative bacteria, indicating poor hygiene practices among the general population. Additionally, MDR bacteria were more common among the Gram-negative isolates, posing a serious threat to immunocompromised individuals in both hospitals and the community.

Studies show that the longer paper currency remains in circulation, the higher the risk of contamination. For example, Egyptian paper notes minted in 2000 had more bacterial contamination than those minted in 2003 [21]. In contrast, no bacterial growth was found on the plastic notes used in our study. We recommend microbial testing of banknotes and the replacement of contaminated or damaged notes by federal authorities. The use of antimicrobial polymers in banknote production or treating banknote paper with antimicrobial compounds could prevent microbial growth and reduce contamination risks [22].

Our study isolated several potentially harmful bacteria from paper currency, including *K*. *pneumoniae*, a virulent organism that can cause pneumonia and urinary tract infections, *Enterobacter aerogenes*, a nosocomial pathogen causing opportunistic infections, and antibiotic-resistant strains of *Streptococcus* and *Staphylococcus*. *E*. *coli*, though often nonpathogenic, can cause serious foodborne illnesses, while *Salmonella* spp. can lead to salmonellosis [23]. Additionally, *S*. *aureus* can cause a wide range of illnesses, from minor skin infections to life-threatening conditions. The highest bacterial contamination in our study came from currency collected at gas stations, which often feature washrooms, convenience stores, food outlets, and mosques. This suggests the need for greater hygiene education for gas station workers, who could be carriers of multiple pathogens.

A SEM revealed cocci and bacillus-shaped bacteria embedded in EPSs, indicating biofilm formation on the currency notes. To our knowledge, biofilm formation in wallets has not been previously reported. Biofilms are microbial communities encased in an extracellular matrix, which allows them to survive under stressful conditions, such as those found in wallets [24]. Biofilms are highly resistant to antimicrobial agents posing a significant public health risk [25–27].

### 4.1. Study Limitations

Limited Internet access or poor connection may have reduced the number of potential participants. Additionally, self-reported data carry inherent biases, as responses could not be independently verified. This study focused solely on Saudi currency notes that had circulated in the community, excluding new notes from banks. In the experimental section of study, we did not include any fungal isolation media, which would facilitate more fungal stains harboring on the bank notes.

## 5. Conclusion

The KAP regarding the use of contaminated currency notes among this group of Saudi adults were found to be inadequate. Significant differences in knowledge were observed based on gender, education level, and employment status. The attitude toward the daily handling, keeping, and storing of contaminated currency notes was generally unfavorable. Practices related to counting contaminated currency and sanitizing hands afterward also varied between male and female participants. Given the detection of various bacteria, including MDR strains, there is a clear need to raise awareness about the risks associated with contaminated currency notes in Saudi Arabia.

## 6. Recommendations

To reduce the spread of infectious diseases and address unhygienic practices related to contaminated currency notes, raising public awareness about the health risks associated with these notes is crucial. Educating people on the impact of contaminated currency on human health is urgently needed. It is advisable to encourage the use of bank and credit cards more frequently, with minimal reliance on physical currency for daily transactions. Additionally, people should be informed about the services offered by national banks, such as the free exchange of old currency notes for new ones. This service can help reduce the circulation of contaminated notes. Further studies should be done on how many days the microorganisms can survive on paper currencies and if they can multiply on them.

## Figures and Tables

**Table 1:** Association between demographic characteristics and knowledge of Saudi population on the use of contaminated currency notes.

Demographic characteristic	Detecting the most contaminated note (Q 6)			Knowledge of the level of contaminants (88%) on Saudi currency notes (Q 7)			Information regarding contaminated notes (Q 10)		
	Most contaminated currency note	Other currency notes	*p*-value	Yes	No	*p*-value	Yes	No	*p*-value
Gender									
Female	1007 (88.18)	135 (11.82)	0.003^∗^	497 (43.52)	645 (56.48)	0.000^∗^	269 (23.56)	873 (76.44)	0.183
Male	223 (81.68)	50 (18.32)		76 (27.84)	197 (72.16)		72 (26.37)	201 (73.63)	
Age									
18–29	686 (89.21)	83 (10.79)	0.071	250 (32.51)	519 (67.49)	n.s.	181 (23.54)	588 (76.46)	n.s.
30–39	215 (86.00)	35 (14.00)		120 (48.00)	130 (52.00)		61 (24.40)	189 (75.60)	
40–49	173 (83.98)	33 (16.02)		95 (46.12)	111 (53.88)		47 (22.82)	159 (77.18)	
50–59	126 (82.35)	27 (17.65)		89 (58.17)	64 (41.83)		42 (27.45)	111 (72.55)	
> 60	29 (82.86)	6 (17.14)		17 (48.57)	18 (51.43)		10 (28.57)	25 (71.43)	
Education									
Low education level	219 (85.21)	38 (14.79)	0.210	125 (48.64)	132 (51.36)	0.002^∗^	67 (26.07)	190 (73.93)	0.229
High education level	1011 (87.31)	147 (12.69)		448 (38.69)	710 (61.31)		274 (23.66)	884 (76.34)	
Employment									
Yes	309 (84.20)	58 (15.80)	0.045	160 (43.60)	207 (56.40)	0.089	96 (26.16)	271 (73.84)	0.158
No	921 (87.88)	127 (12.12)		413 (39.41)	635 (60.59)		245 (23.38)	803 (76.62)	

Abbreviation: n.s., not significant.

^∗^Significant *p*-value (*p* < 0.05).

**Table 2:** Practice of Saudi population on the use of contaminated currency notes.

Practice of Saudi population on the use of contaminated currency notes	Frequency (%)
	*N* = 1415
What do you wash your hand with:^∗^, ^∗∗^	
Water	47 (7.73)
Water and soap	529 (87.01)
Liquid sterile	173 (28.45)
Wipes sterile	120 (19.73)
Plain tissue paper	6 (1)
Any other, please specify: perfume	2 (0.33)
If you are given a bundle of 50 currency notes in riyals, how would you count them?	
I will use my saliva on the tip of my finger	148 (10.97)
I will wet my finger in water and start counting	39 (2.90)
I will separate the notes with my finger only	1165 (86.49)
Any other method used, please specify:^∗∗∗^	
Counting machine	38 (2.68)
Using lotion cream	4 (0.28)
Wet sponge	5 (0.35)
Wearing gloves	5 (0.35)
Put them on the floor and count	7 (0.49)
Giving the salesman for counting	1 (0.1)
Disinfectant the currency	3 (0.21)
How do you store currency notes in general, please explain?^∗∗∗^	
Wallet	1082 (76.39)
Bags	134 (9.46)
Inside clothes pocket	88 (6.21)
Treasury	59 (4.16)
Drawer	54 (3.81)
Other (under couch, chair, carpet, inside file and books and mobile cover between makeup products)	46 (3.25)
Inside envelope	37 (2.61)
In the bank	31 (2.19)
Closet/cabinet	26 (1.83)
Credit card	12 (0.84)
In dry and safe place	8 (1)

^∗^Permit more than one answer for each question.

^∗∗^Out of 608 respondents.

^∗∗∗^Open questions.

**Table 3:** Attitude and practice of Saudi population on the use of contaminated currency notes.

Attitude and practice of Saudi population on the use of contaminated currency notes:	Frequency (%)
	*N* = 1415
What do you think about keeping and storing currency notes under carpet, or while wearing: inside the clothes, in socks, and inside shoes	
It is an acceptable behavior	133 (9.40)
It is not an acceptable behavior	1282 (90.60)
In your opinion, what is the best place to store and keep the currency notes other than the bank, please explain:^∗^	
Treasury	365 (25.79)
Closet/cabinet	136 (9.61)
Boxes	120 (8.48)
Bags	79 (5.58)
Drawer	61 (4.31)
Envelope	55 (3.88)
At home	54 (3.81)
Moneybox	47 (3.32)
I do not know	33 (2.33)
Wallet	29 (44.45)
In a safe and clean place and exposure to sun light, away from dust, pollutants, moisture, and children	20 (1.41)
Others (under pillow, carpet and bed, and inside tissues, books, file, and album)	19 (1.34)
Clothes pocket	17 (1.20)
Master card	8 (1)
Bit-coin	1 (0.07)

^∗^Open question.

**Table 4:** Association between demographic characteristic and attitude Saudi population on the use of contaminated currency notes.

Demographic characteristic	What to do with contaminated currency notes? (Q 11)			Using saliva to count a bundle of currency notes? (Q 15)			Keeping and storing currency notes under carpet, or while wearing: Inside the clothes, in socks, and inside shoes? (Q 17)		
	Yes	No	*p*-value	It is a good and a widespread practice	It is not good and unhealthy practice	*p*-value	It is an acceptable behavior	It is not an acceptable behavior	*p*-value
Gender									
Female	163 (12.27)	979 (85.73)	0.021^∗^	23 (2.01)	1119 (97.99)	0.000^∗^	86 (7.53)	1056 (92.47)	0.000^∗^
Male	26 (9.52)	247 (90.48)		26 (9.52)	247 (90.48)		47 (17.22)	226 (82.78)	
Age									
18–29	26 (7.28)	713 (92.72)	n.s.	28 (3.64)	741 (96.36)	0.225	84 (10.92)	685 (89.08)	n.s.
30–39	45 (18.00)	205 (82.00)		8 (3.20)	242 (96.80)		23 (9.20)	227 (90.80)	
40–49	36 (17.48)	170 (82.52)		11 (5.34)	195 (94.66)		16 (7.77)	190 (92.23)	
50–59	44 (28.76)	109 (71.24)		2 (1.31)	151 (98.69)		7 (4.58)	146 (95.42)	
> 60	8 (22.86)	27 (77.14)		0 (0.00)	35 (100.00)		2 (5.71)	33 (94.29)	
Education									
Low education level	40 (15.56)	217 (84.44)	0.147	11 (4.28)	246 (95.72)	0.265	23 (8.95)	234 (91.05)	0.446
High education level	149 (12.87)	1009 (87.13)		38 (3.28)	1120 (96.72)		110 (9.50)	1048 (90.50)	
Employment									
Yes	63 (17.17)	304 (82.83)	0.009^∗^	19 (5.18)	348 (94.82)	0.03^∗^	24 (6.54)	343 (93.46)	0.016^∗^
No	126 (12.02)	922 (87.98)		30 (2.86)	1018 (97.14)		109 (10.40)	939 (89.60)	

Abbreviation: n.s., not significant.

^∗^Significant *p*-value (*p* < 0.05).

**Table 5:** Association between demographic characteristics and practice of the Saudi population on the use of contaminated currency notes.

Demographic characteristic	Wash hands after handling contaminated notes (Q 12)			Method of counting notes (Q 13)			
	Yes	No	*p*-value	Use saliva	Wet fingers	Only with fingers	*p*-value
Gender							
Female	547 (47.90%)	595 (52.10%)	0.000^∗^	102 (9.32%)	33 (3.02%)	959 (87.66%)	0.005^∗^
Male	60 (21.98%)	213 (78.02%)		41 (16.21%)	6 (2.37%)	206 (81.42%)	
Age							
18–29	229 (29.78)	540 (70.22)	n.s.	54 (7.34)	2 (0.27)	680 (92.39)	n.s.
30–39	130 (52.00)	120 (48.00)		37 (15.42)	8 (3.33)	195 (81.25)	
40–49	121 (58.74)	85 (41.26)		27 (13.85)	10 (5.13)	158 (81.03)	
50–59	105 (68.63)	48 (31.37)		19 (13.48)	17 (12.06)	105 (74.47)	
> 60	21 (60.00)	14 (40.00)		6 (18.18)	2 (6.06)	25 (75.76)	
Education							
Low education level	120 (46.69)	137 (53.31)	0.098	39 (15.98)	17 (6.97)	188 (77.05)	n.s.
High education level	487 (42.06)	671 (57.94)		104 (9.43)	22 (1.99)	977 (88.58)	
Employment							
Yes	183 (49.86)	184 (50.14)	0.001^∗^	51 (14.41)	17 (4.80)	286 (80.79)	0.000^∗^
No	424 (40.46)	624 (59.54)		92 (9.26)	22 (2.22)	879 (88.52)	

Abbreviation: n.s., not significant.

^∗^Significant *p*-value (*p* < 0.05).

**Table 6:** Distribution of Gram-negative bacteria on different currency notes.

Microorganisms	Currency						Total	MDR (%)
	1	5	10	50	100	500		Positive
*Acinetobacter* spp.	0	2	1	0	2	1	7	4 (57%)
*Aeromonas hydrophila* complex	1	0	0	0	0	1	2	1 (50%)
*Citrobacter* spp.	0	0	0	1	0	1	2	1 (50%)
*Cupriavidus pauculus*	0	0	0	0	0	1	1	1 (100%)
*Enterobacter* spp.	1	0	1	2	2	2	8	8 (100%)
*Escherichia coli*	2	4	4	1	2	2	15	12 (80%)
*Vibrio metschnikovii*	0	1	0	0	0	0	1	1 (100%)
*Klebsiella* spp.	0	2	1	1	1	1	6	6 (100%)
*Kluyvera intermedia*	0	0	0	0	1	0	1	0 (0%)
*Pantoea agglomerans*	0	0	0	0	2	1	3	2 (66%)
*Pseudomonas aeruginosa*	3	0	2	0	0	0	5	2 (40%)
Other *Pseudomonas* spp.	1	0	1	0	2	1	5	2 (40%)
*Rhizobium radiobacter*	0	0	0	0	1	0	1	1 (100%)
*Stenotrophomonas maltophilia*	0	0	0	0	0	1	1	0 (0%)
*Yersinia pseudotuberculosis*	1	1	0	0	0	0	2	0 (0%)
Total (MDR%)	10 (20%)	10 (70%)	10 (90%)	5 (100%)	13 (76%)	12 (66%)	60	41 (68%)

**Table 7:** Distribution of Gram-positive bacteria on different currency notes.

Microorganism	Currency						Total	MDR (%)
	1	5	10	50	100	500		Positive
*E*. *faecalis*	0	2	1	0	1	0	4	4 (100%)
*E*. *faecium*	0	0	0	0	1	0	1	1 (100%)
*S*. *aureus*	0	2	1	0	0	1	4	4 (100%)
*S*. *auricularis*	0	0	0	1	0	2	3	3 (100%)
*S*. *haemolyticus*	0	1	0	0	0	0	1	1 (100%)
*S*. *hominis* subsp. *novobiosepticus*	1	1	1	1	0	1	5	4 (80%)
*S*. *intermedius*	0	0	0	0	0	1	1	1 (100%)
*S*. *sciuri*	1	0	0	0	0	0	1	1 (100%)
*S*. *xylosus*	0	0	1	0	0	2	3	3 (100%)
*S*. *agalactiae* non-hemolytic	1	0	0	0	0	0	1	0 (0%)
Total (MDR%)	3 (33.3%)	6 (100%)	4 (100%)	2 (100%)	2 (100%)	7 (100%)	24	22 (91.6%)

**Table 8:** Location and currency-wise distribution in Gram-negative and Gram-positive microorganisms.

Location	Gram-negative organisms								Gram-positive organisms							
	Currency						Total	MDR (%)	Currency						Total	MDR (%)
	1	5	10	50	100	500			1	5	10	50	100	500		
Food refrigerators	1	0	1	1	4	4	11	54.5%	0	0	1	0	0	0	1	100%
Gas stations	4	7	2	1	2	1	17	70.6%	2	2	1	0	1	2	8	75%
Grocery shop	0	1	2	1	1	2	7	100%	0	0	1	2	1	1	5	100%
Hospital cafeteria	1	0	0	0	1	0	2	100%	0	2	1	0	0	3	6	100%
Restaurants	0	2	3	2	3	2	12	91.7%	0	1	0	0	0	1	2	100%
Slaughterhouses	2	0	0	0	0	0	2	0%	1	1	0	0	0	0	2	100%
Vegetable store	2	0	2	0	2	3	9	44.4%	NA							
Total	10	10	10	5	13	12	60	68.3%	3	6	4	2	2	7	24	91.6%

Abbreviation: NA, not available.

## Data Availability

The data that support the findings of this study are in this published article and available from the corresponding author upon reasonable request.

## Supporting Information

Online survey/questionnaire (web-based survey): Online survey invitations were published in the form of a URL link on websites or in other media, which leads to data collection by snowball sampling method.
