# Supplementary material for: Knowledge, Attitudes, and Practices of the Saudi Arabian Population Regarding Contaminated Banknotes: Implications for Infectious Disease Transmission and Analyzing the Biofilm in Wallet as a Reservoir
Source: Can J Infect Dis Med Microbiol. 2025 May 22;2025:4611971. doi: 10.1155/cjid/4611971 (PMC12122151; doi:10.1155/cjid/4611971)
Supplement: Supporting Information — Additional supporting information can be found online in the Supporting Information section. [file 4611971.f1.docx]

**Appendix 1**

**SURVEY QUESTIONNAIRE**

**Questionnaire ID Number: ___**

**Please answer the following questions to the best of your knowledge and ability.**

**1-Please indicate your gender:**

**1=Female 2= Male**

**2-What is your current age (in years): ____________**

**3-In which city do you currently live: ______________________________**

**4-What is your education level:**

**1= literacy 3= post-graduated**

**2= High school 4= Professional**

**3= University**

**5-Are you currently employed?**

**1= Yes**

**2= No**

**If Yes, please state your occupation: _______________________**

**6-In your opinion which is the most contaminated currency note among the four pictured below? ( in general , the contamination not restricted on riyal currency note )**

**A:**

**
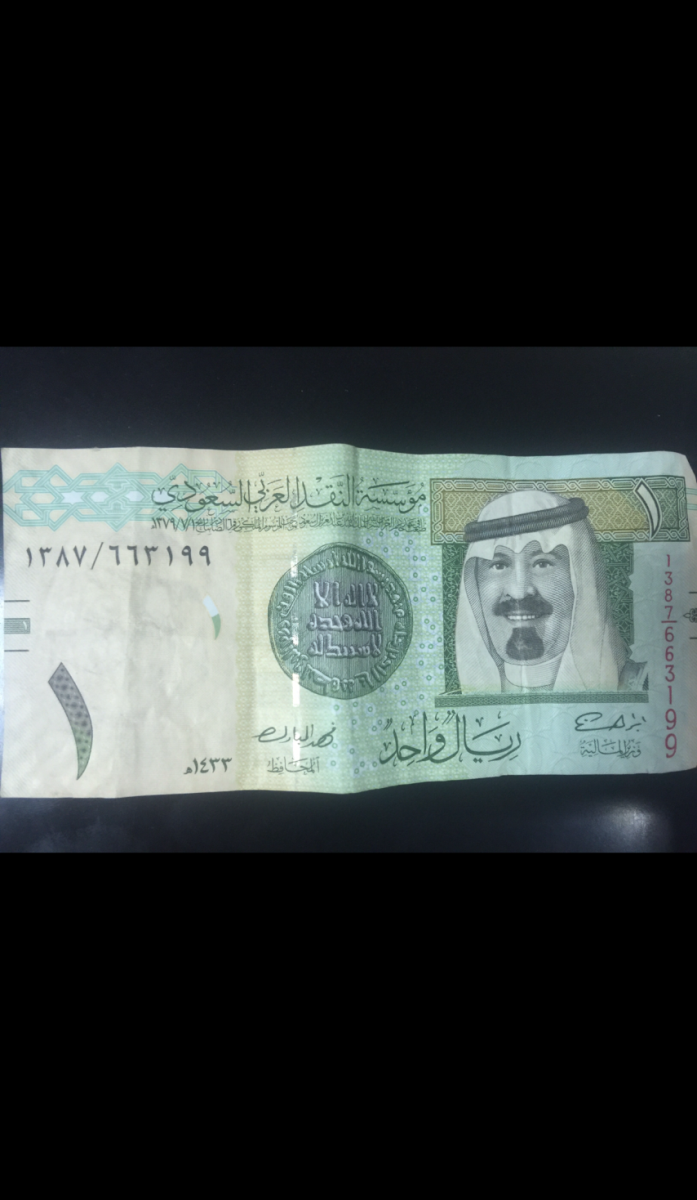
**

**B:**

**
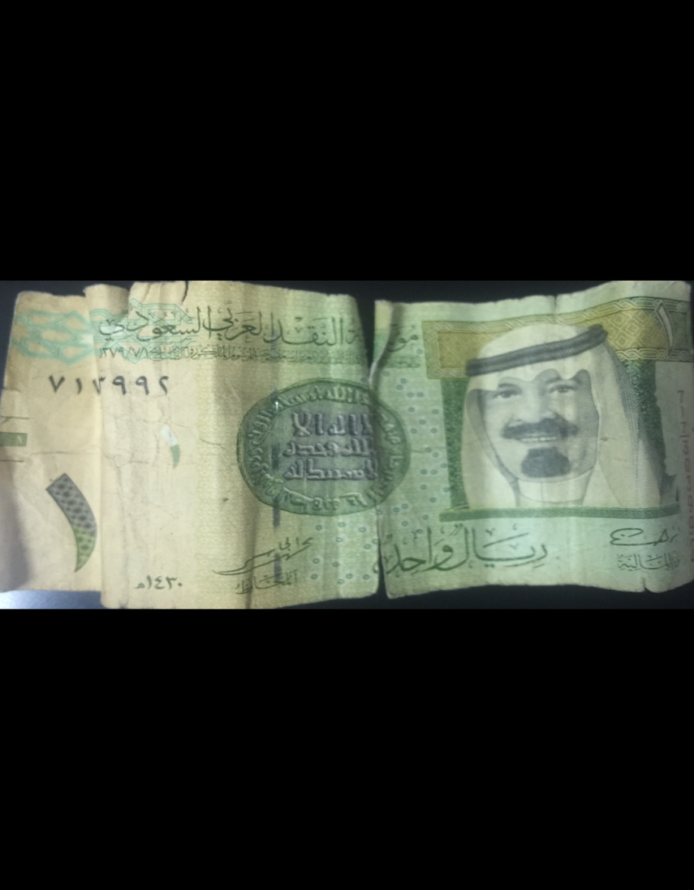
**

**C:**

**
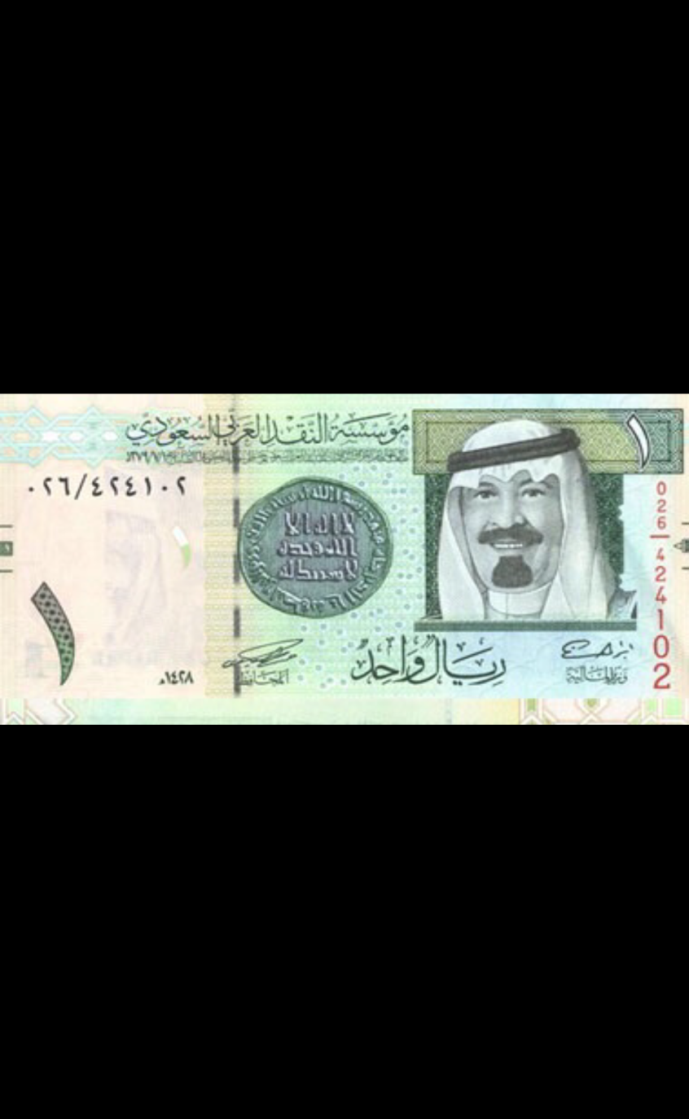
**

**D:**

**
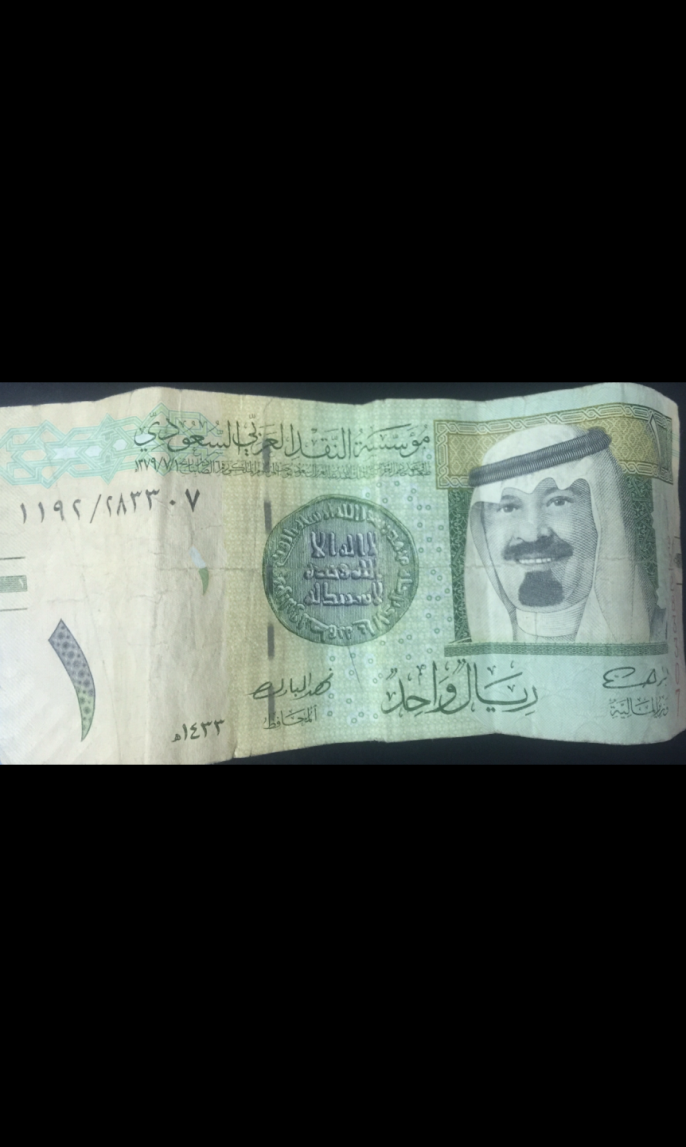
**

**7-Do you know the level of contaminants on Saudi currency notes reach 88 % ?**

**1= yes**

**2 = no**

**8-Where the place do you think you will get from it the most contaminated currency notes?**

**(you may choose more than one options that may apply to the question)**

**1= gas station 5= meat shop**

**2= grocery shop**

**3= cafeteria in the hospital**

**4=cafeteria in the school, office and collage**

**6= restaurant**

**7= farm market ( the products sold by farms )**

**9-What are the probable contaminants do you think are on the currency notes?**

**(you may choose more than one options that may apply to the question)**

**1= microbes 5= drinks (e.g., coffee, tea, juices, water, soda ,etc.)**

**2= dust 6= hand sweat**

**3= dirt 7= Any other, please specify: ______________________**

**4=food particles**

**10- did you receive information regarding contaminated currency notes?**

**1= yes**

**2= no**

**If yes , from where did you receive information regarding contaminated currency**

**notes :**

**_________________________________________________________________________**

**11-Do you know what to do with contaminated currency notes?**

**1= yes**

**2= no**

**If Yes, what do you do with contaminated currency notes when you receive it:**

**__________________________________________________________________**

**12-Would you wash your hand after handling contaminated currency notes?**

**1= yes**

**2= no**

**If Yes, what do you wash your hands with: _____________________________________**

**1= water**

**2= water and soap**

**3=liquid sterile**

**4= wipes sterile**

**5=plain tissue paper**

**6=Any other, please specify: ___________________________________**

**13-If you are given a bundle of 50 currency notes in Riyals, how would you count them?**

**
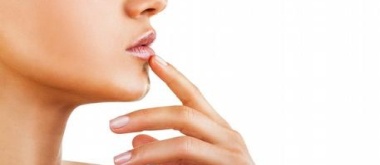
 1= I will use my saliva on the tip of my finger.**

**
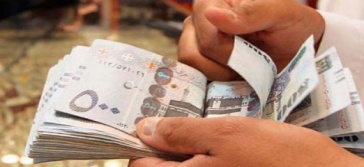
 2= I will wet my finger in water and start counting.**
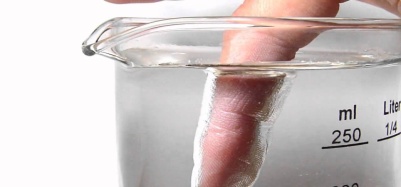
 **3= I will separate the notes with my finger only.**

**4= Any other method used, please specify: ___________________________________**

**14- How do you store currency notes in general, please explain?**

**_______________________________________________________________________**

**15-What do you think about using saliva when counting a bundle of currency notes ?**

**1= It is good and a widespread practice.**

**2= It is not good and is an unhealthy practice.**

**16- (gastroenteritis or inflammation in intestine , sore throat , acne , blisters , diarrhea , allergy in the eye ) Do you think their sources from contaminated currency notes ?**

**1= yes**

**2= no**

**17-What do you think about keeping and storing currency notes under the carpet, or while wearing: inside the clothes; in socks; and inside shoes?**

**1= It is an acceptable behavior**

**2= It is not an acceptable behavior**

**18-In your opinion, what is the best place to store and keep the currency notes other than the bank, please explain?**

**_____________________________________________________________________**

**19- In your opinion, does Saudi population needs an awareness and knowledge about contaminated currency notes ?**

**1= yes**

**2= no**

***Thank you for taking the time to complete this questionnaire.***

**Appendix 2**

| List of antibiotics tested for Gram positive bacteria | List of antibiotics tested for Gram negative bacteria |
| --- | --- |
| Amox/k Clav | Amikacin |
| Ampicillin | Amox/k Clav |
| Azithromycin | Amp/Sulbactam |
| Cefoxitin Screen | Ampicillin |
| Ciprofloxacin | Aztreonam |
| Clindamycin | Cefazolin |
| Daptomycin | Cefepime |
| Erythromycin | Cefotaxime |
| Fosfomycin | Cefotaxime/K Clavulanate |
| Fusidic Acid | Cefoxitin |
| Gentamicin | Ceftazidime |
| Imipenem | Ceftazidime/K Clavulanate |
| Levofloxacin | Cefuroxime |
| Linezolid | Ciprofloxacin |
| Moxifloxacin | Colistin |
| Mupirocin | Ertapenem |
| Nitrofurantoin | Gentamicin |
| Oxacillin | Imipenem |
| Penicillin | Levofloxacin |
| Rifampin | Meropenem |
| Synercid | Moxifloxacin |
| Teicoplanin | Nitrofurantoin |
| Tetracycline | Norfloxacin |
| Trimeth/Sulfa | Pip/Tazo |
| Vancomycin | Tigecycline |
|  | Tobramycin |
|  | Trimeth/Sulfa |
